# Metal Concentrations in Edible Leafy Vegetables and Their Potential Risk to Human Health

**DOI:** 10.3390/ijerph23020188

**Published:** 2026-01-31

**Authors:** Elizabeth Kola, Linton F. Munyai, Caswell Munyai, Sydney Moyo, Farai Dondofema, Naicheng Wu, Tatenda Dalu

**Affiliations:** 1 Aquatic Systems Research Group, School of Biology and Environmental Sciences, University of Mpumalanga, Nelspruit 1200, South Africa; linton.munyai@ump.ac.za (L.F.M.);; 2 College of Agriculture, Engineering and Science, University of Kwa Zulu Natal, Scottsville 3209, South Africa; munyaic2@ukzn.ac.za; 3 Department of Biological Sciences, Louisiana State University, Baton Rouge, LA 70803, USA; sydmoyo@gmail.com; 4 South African Institute for Aquatic Biodiversity, Makhanda 6140, South Africa; farai.dondofema@univen.ac.za; 5 Department of Geography and Spatial Information Techniques, Ningbo University, Ningbo 315211, China; wnaicheng@gmail.com

**Keywords:** bioaccumulation, carcinogenic, health risk, heavy metals, leafy vegetables

## Abstract

Leafy green vegetables provide important nutrients for human growth; however, human health is highly compromised through consumption of vegetables contaminated by heavy metals. Therefore, the study aimed to investigate the bioaccumulation of heavy metals in five different leafy green vegetables and soils and determine the human health risks that may arise from consuming those vegetables from Tonga town in Mpumalanga province, South Africa. Soils and five edible leafy vegetables (i.e., lettuce, cabbage, rape, pumpkin leaves, and spinach) were assessed for bio-concentration factor, daily intake of metals, health risk, and target hazard quotient across the study sites. The Si, K, Na, Ca, Mg, Al, and Fe concentrations were high in the soils. In general, vegetables exhibited elevated Ca, Fe, Si, Al, and Sr levels, although spinach had high Na concentrations. The bioconcentration factor showed the following trends: Mg > B > Si > V for trace metals and Cr > Co > Mn > Ni > B for heavy metals in lettuce, spinach, and pumpkin leaves. The human risk index for all vegetables showed that all metals were not likely to induce any health hazards to humans, and the target hazard quotient for B, Si, V, Al, Cr, Mn, Fe, Ni, Zn, and Pb showed potential for substantial health risk hazard. The findings of this study generally reveal that the concentrations of the analysed metals exceeded the permissible limits established by the World Health Organisation and the Food and Agricultural Organisation. Given the high levels of metals detected in the soil and vegetables within the study area, it is important to investigate the potential implications for human health and mitigate both acute and chronic health challenges associated with heavy metal exposure. Furthermore, this study will guide policymakers in developing improved regulations and safety standards for agricultural practices and environmental protection, particularly for vulnerable peri-urban and rural communities.

## 1. Introduction

Metal contamination in leafy vegetables has become an increasing concern due to the potential risk it poses to human health [1]. Leafy edible vegetables, such as spinach, cabbage, cauliflower, Swiss chard, rape, tomato, lettuce, and kale, are widely consumed due to their high nutritional value [1,2]. However, they can also accumulate toxic metals such as lead (Pb), cadmium (Cd), arsenic (As), copper (Cu), and mercury (Hg) from contaminated soils and water [3,4,5,6]. This contamination can occur through multiple pathways, including agricultural practices involving the application of pesticides, herbicides, fertilisers, wastewater irrigation, and proximity to urban or mining areas [7,8,9]. The accumulation (bioaccumulation) of metals in leafy vegetables presents a significant risk, as these metals can be transferred to animals and humans through consumption [10,11,12]. Certain metals such as zinc (Zn) and magnesium (Mg) are essential for human growth, but in small quantities. However, excessive exposure to these metals can lead to adverse health effects [13,14,15]. A notable example is that of cadmium (Cd), which can cause kidney damage and bone complications [16,17]. Lead (Pb) is known to negatively affect cognitive development in children and cause cardiovascular problems in adults, [18,19], due to its carcinogenic effects [20,21], and mercury (Hg) toxicity can lead to neurological disorders [22,23].

The African continent is experiencing rapid urbanisation and industrialisation, which has led to increased pollution and the contamination of water and soil. Many farming activities, human activities, and climate change, especially in urban and peri- [24,25,26]. Many farming activities [27,28], human activities and climate change [29] especially in urban and peri–urban areas, occur near industrial zones, floodplain areas, wetlands and/or along highways, exposing vegetables to environmental pollutants. These farming activities often rely on irrigation from potentially contaminated water sources and contaminated soils [30,31,32], and contaminated soils [33] increasing the likelihood of metal uptake by crops. Therefore, understanding metal concentrations in these vegetables is essential to adhere to the developed guidelines for safe consumption levels and potential threats to animal and human health. Some metals are suitable for human consumption. However, they must not exceed the permissible limits described by the World Health Organisation (WHO) [34]. Regular dietary intake of contaminated vegetables could result in gradual bioaccumulation in the human body, leading to chronic health issues [35,36]. 

Vulnerable communities, especially those in rural areas that depend on subsistence farming are at risk, making it imperative to assess the potential dangers of consuming contaminated vegetables. Hence, the current study aimed to evaluate metal concentrations in vegetables cultivated and sold by subsistence farmers in the town of Tonga in the Ehlanzeni District Municipality of Mpumalanga in South Africa. In addition, we assess the non-carcinogenic risk of vegetable consumption on human health in a rural area in the subtropical South African region. Thus, we anticipate this research will guide policymakers in developing improved regulations and safety standards for agricultural practices and environmental protection, particularly for vulnerable peri-urban and rural communities. Doing so will help safeguard public health and promote sustainable food production in South Africa.

## 2. Materials and Methods

### 2.1. Study Site

**Figure 1:**
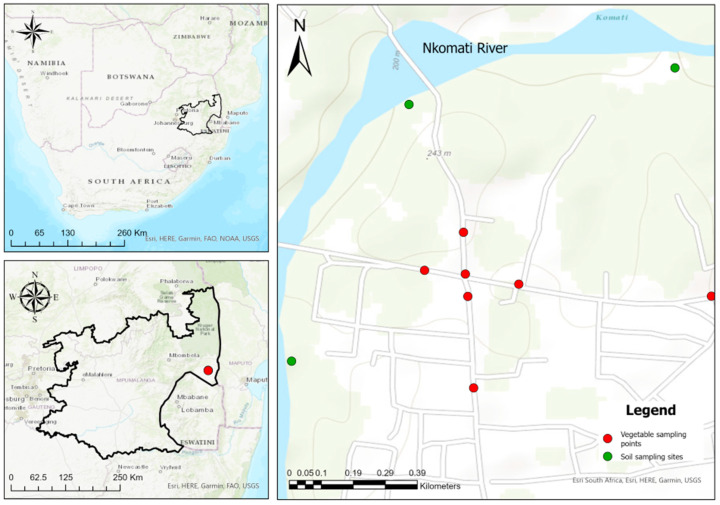
Location of the study area in Tonga, Nkomazi Municipality, Mpumalanga Province of South Africa.

Samples were collected from Tonga (25.7097′ S, 31.7195′ E), Nkomazi Local Municipality in the Mpumalanga province of South Africa (Figure 1). This municipality covers 4786.86 km^2^, which is approximately 17% of the land of the Ehlanzeni district (Nkomazi Local Municipality, 2020). This municipality has 43 villages, and Tonga is one of them (Nkomazi Local Municipality, 2020). Tonga was the site of interest because they use water from the wetlands to irrigate their leafy vegetables.

Tonga is a town in the Nkomazi Local Municipality, which is dominated mainly by Shangaan/Tsonga and SiSwati speaking ethnic groups due to its closeness to Mozambique and Eswatini [37]. Tonga’s population was 17,333 as of the 2011 census and is estimated to reach over 591,000 residents by 2022. In 2011, the census reported Tonga’s population as having a relatively high density of about 2300 people per square kilometer [38]. On average, summer temperatures can reach 35 °C or higher during heatwaves, whereas winter can be 10 °C. This area receives summer rainfall between 750 mm and 860 mm, while winters are generally dry.

Geologically, Tonga is based on the Kaapvaal Craton, identified by granite, gneiss, and basalt, impacting the region’s soil quality. This combination of geological features and soil types (clay, sandy, and alluvial soils) supports agricultural activities, especially sugarcane farming. Alluvial soil is naturally found along the riverbanks, which supports most agricultural production [39]. The main biome within Tonga is the savanna, which forms one-third of South Africa. It is characterised by an upper covering of shrubs, scattered trees, and a ground covering of grasses [40]. 

### 2.2. Collection of Vegetables and Soils

Edible leafy vegetables (i.e., lettuce, cabbage, rape, pumpkin leaves, spinach, *n* = 10–40 leaves per vegetable type) were randomly purchased from different traders within Tonga in September 2023. Immediately after each purchase, each leafy bundle (*n* = 10) was placed individually in a labelled Ziplock bag and transported to the laboratory. Prior to purchasing the vegetables, the traders were asked where they got the vegetables from; furthermore, visitation of the cultivation areas/gardens was conducted to confirm the source of irrigation water, which was from the nearby river system (i.e., Nkomati). These edible leafy vegetables were not deposited in the herbarium, they are common local vegetables within the study area. Integrated soil samples (*n* = 2500 g) from different sites were collected using a plastic hand shovel and placed into 164 polyethylene Ziplock bags.

### 2.3. Vegetable and Soil Sample Preparation

In the laboratory, the vegetables were washed with distilled water to remove any soil particles. Each vegetable leaf type was then oven-dried at 60 °C for 72 h. After drying, soil samples were ground using a porcelain pestle and mortar and sieved through a 63 μm sieve to remove any plant material. The dried vegetables were ground into a fine powder and homogenised using a kitchen blender (Summit^®^ Pro Bar Blend series BBS1200, Hamilton Beach Brands Inc., Glen Allan, VA, USA) and 20 g of powder of each vegetable was weighed and placed into an airtight Ziplock bag for the determination of metal concentrations: magnesium (Mg), calcium (Ca), sodium (Na), potassium (K), phosphorus (P), boron (B), aluminium (Al), silicon (Si), vanadium (V), chromium (Cr), manganese (Mn), iron (Fe), cobalt (Co), nickel (Ni), copper (Cu), zinc (Zn), arsenic (As), molybdenum (Mo), cadmium (Cd), tin (Sn), barium (Ba), strontium (Sr), and lead (Pb). To determine metal concentrations, 0.5 g of each powdered vegetable was weighed and transferred into a Teflon digestion vessel, where 7 mL of high-purity concentrated 65% nitric acid (HNO_3_) and 1 mL of high-purity concentrated 30% hydrogen peroxide (H_2_O_2_) were added. Subsequently, the vessels were capped, fitted into rotor segments, and inserted into the microwave digestion system, where the samples were radiated for 20 min. Upon cooling, the solution was transferred into a 50 mL volumetric flask before analysis using an Inductively Coupled Plasma Mass–Spectrophotometer (ICP–MS) instrument with an Elan 9000 (Perkin Elmer, Waltham, MA, USA) and Agilent 7500a (Agilent, Santa Clara, CA, USA) [6]. All sample analyses were conducted at the Central Analytical Facility, Stellenbosch University, South Africa. The accuracy % of the internal quality control ranged from 89.4% to 113.6%, with the tomato leaf certified reference material having a recovery % on certified reference of 86.2% to 114.4%.

### 2.4. Data Analysis

#### 2.4.1. Statistical Analysis 

The data were first tested for normality (i.e., using Shapiro–Wilk test) and 203 homogeneity of variances (i.e., Levene’s test) and were found to meet all assumptions of parametric analysis. A one–way analysis of variance (ANOVA) was used to determine the differences in metal concentrations among vegetable types (i.e., lettuce, cabbage, rape, pumpkin leaves, spinach) using SPSS version 25. A principal component analysis (PCA) with the varimax rotation method was used to determine the natural and/or anthropogenic sources of sediment metals among vegetable types, was used for metal source identification in PC–ORD version 5.10.

#### 2.4.2. Bio-Concentration Factor of Metals

Bio-concentration factor (BCF) of metals translocated from the soils to vegetables was measured by calculating the ratio of the concentration of each metal in vegetable leaves and the concentration of the corresponding metal in the respective soil where these vegetables were grown [41]. A BCF value of less than 1 means minimal translocation of metals from the soils to vegetables, and a value greater than 1 means a high translocation of metals from soils to vegetables.

#### 2.4.3. Daily Intake of Metals

The daily intake of metals (DIM) is the potential risk associated with the ingestion of heavy metals via the consumption of vegetables [42] and was calculated using the following equation:

$$DIM=\frac{C_{metal}\times C_{factor}\times D_{food\;intake}}{B_{average\;weight}}$$

where, C_metal_ represents the metal concentrations in vegetables (mg/kg^−1^), C_factor_ is a conversion factor which is estimated at 0.085 [42], the human average body weight (B_average weight_) in South Africa is 60.7 kg [43], and the intake of vegetables (D_food intake_) for South Africans is estimated at 200 g per person per day [44,45].

#### 2.4.4. Health Risk Index

The risk to human health (HRI) by intake of metal through the consumption of vegetables in the study area was calculated using an equation described by the following equation [6]:

HRI=DIM/RfD

where DIM is the daily exposure to metals and RfD is the reference oral dose. The RfD values used in this study for boron (B), aluminium (Al), silicon (Si), vanadium (V), chromium (Cr), manganese (Mn), iron (Fe), cobalt (Co), nickel (Ni), copper (Cu), zinc (Zn), arsenic (As), molybdenum (Mo), cadmium (Cd), tin (Sn), barium (Ba), and lead (Pb) are 0.2, 1, 0.005, 0.007, 1.5, 0.033, 0,7, 0.02, 0,02, 0,4, 0.003, 0.005, 0.6, 0.004, 0.2, 0.004, respectively [2,46,47]. If the ratio is lower than 1, then vegetables are not likely to induce any health hazard to humans, and if the HRI value is equal to or higher than 1, it is considered to be not safe for humans, as a potential health risk may occur [48]. 

#### 2.4.5. Target Hazard Quotient

The target hazard quotient (THQ) assesses the non-cancerous risks due to heavy metal intake through regular intake of contaminated vegetables [36]. The health risk hazard is enhanced with increased THQ and was calculated using the following equation:

$$THQ=\frac{EDI}{\mathrm{RfD}}$$

where EDI refers to estimated daily intake of heavy metals (mg/person/day), and RfD is oral reference dose. THQ values of less than 1 mean no non-carcinogenic risks, and values greater than 1 indicate there is potential for a substantial health risk hazard.

## 3. Results

### 3.1. Soil and Vegetable Trace and Heavy Metal Dynamics

#### 3.1.1. Soil Trace Metal Concentrations

The mean concentrations of trace metals in the soil were generally high for Si, K, Na, Ca, and Mg. In contrast, lower concentrations were observed for P, V, and B (Table 1). Similarly, for heavy metals in the soil, elevated levels were recorded for Al and Fe, while much lower concentrations were measured for Cu, Ni, Pb, Co, As, Cd, and Hg (Table 1).

#### 3.1.2. Trace Metal Concentrations in Vegetables

Cabbage exhibited notably high Ca, K, Na, Mg, and P concentrations, while low concentrations were observed for Si, B, and V (Table 1). Rape showed elevated concentrations of Ca, K, Na, Mg, and P, whereas low concentrations were recorded for Si, B, and V, indicating a similar trend as observed in cabbage (Table 1). On average, trace metal concentrations in lettuce were high in K, Ca, Na, P, and Mg, with distinctly lower concentrations of Si, B, and V (Table 1). Concentrations of K, Ca, P, and Mg characterised pumpkin leaves. However, the concentrations of Na, Si, B, and V were generally lower in pumpkin leaves. Spinach leaves exhibited high Na, K, Mg, Ca, and P concentrations, coupled with low concentrations of Si, B, and V (Table 1). An Analysis of Variance (ANOVA) revealed significant differences (*p* < 0.05) in the concentrations of Na, Mg, P, K, and Ca (Table 2).

#### 3.1.3. Heavy Metal Concentrations in Vegetables

For heavy metals, cabbage exhibited high concentrations of Fe, Si, Al and Sr, with low concentrations being observed for metals such as Ba, Cr, Ni, Mo, Sn, Pb, Cd, As, and Hg (Table 1). In rape, heavy metal concentrations were high in Sr, Fe, and Al, while low concentrations were recorded for Ni, Mo, Sn, and Co (Table 1). Heavy metals such as As, Cd, Pb, As, and Hg were below the 0.1 mg kg^−1^ permissible level (Table 1). Lettuce had high heavy metal concentrations of Al and Fe, with lower levels in Co, Sn, Mo, Pb, As, Cd, and Hg (Table 1). The mean concentration of heavy metals in pumpkin leaves was generally high for Al and Fe, with low concentrations recorded for Co, Mo, Sn, Pb, As, Cd, and Hg (Table 1). Spinach leaves were characterised by high levels of Al and Fe, with low concentrations of Co, Sn, Mo, Pb, As, Cd, and Hg (Table 1). Analysis of variance for various metals in leafy edible vegetables indicated that Al, Fe, Cu, Zn, As, Sr, Mo, and Cd showed significant differences (*p* < 0.005) among vegetable groups (Table 2), indicating variability in heavy metal accumulation across the studied vegetables (Table 2).

### 3.2. Variation and Relationships of Metals in Vegetables

**Figure 2:**
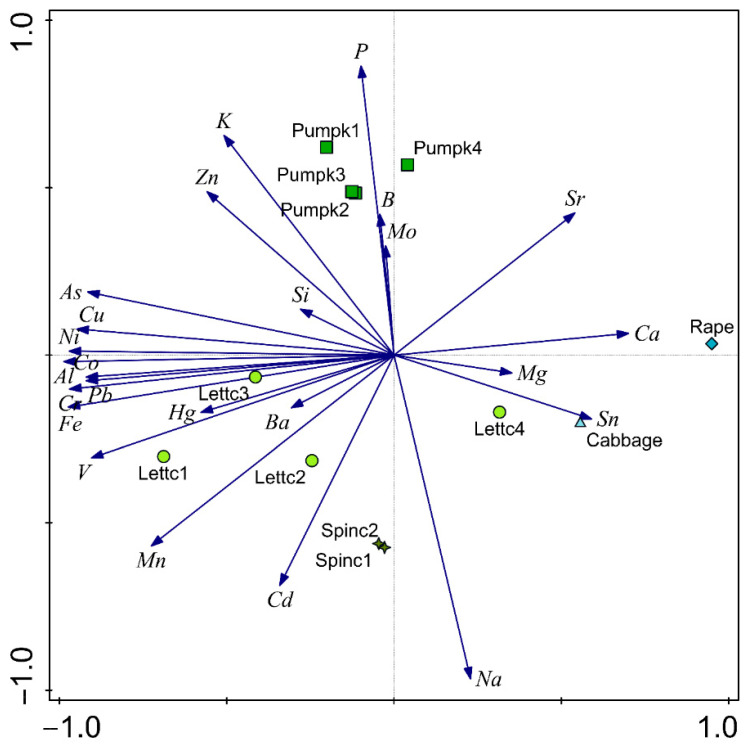
The principal component plot in rotated space for trace and heavy metals in the study area. Abbreviations for edible leafy vegetables: Spinc—spinach, Lettc—lettuce, Pumpk—pumpkin leaves.

Our data revealed considerable variation and relationships across different species, reflecting differences in their uptake and accumulation capacities (Figure 2). Principal component analysis (PCA) results (Table 3) revealed that axis 1 accounted for 42.5% of the total variance. This axis showed high and moderate positive relations for Sr, Ca, Mg, and Sn and high negative relations for V, Al, Cr, Mn, Fe, Co, Ni, Cu, As, Hg, and Pb (Figure 2; Table 3). Axis 2 explained 18.1% of the total variance with positive and significant loadings for P, K, B, and Zn and high negative relations for Na and Sn (Table 3). The PCA biplot indicated two distinct metal components, showing the clustering of heavy and trace metals. Metals including As, Cu, Ni, Co, Al, Pb, Ca, Fe, Pb, and Fe clustered with Lettc3. Further, the plot indicates that V had a strong relationship with Lettc1, Mn with Lettc2, and Cd with Spinc2 and Spinc1. A negative relationship between Ba and other metals was observed, where increasing concentrations of other metals corresponded to a decrease in Ba. In terms of P, it clustered with all four pumpkin leaves. The results suggest that concentrations of B and Mo in pumpkins could be lowered with increasing P, and K and Zn could lower Si in pumpkins. The Ca had a strong association with rape, and Sn with cabbage. It is clear to note that Sn in cabbage and lettuce will be lowered with an increase in Na.

### 3.3. Health Indices

#### 3.3.1. Bio-Concentration Factors

**Figure 3:**
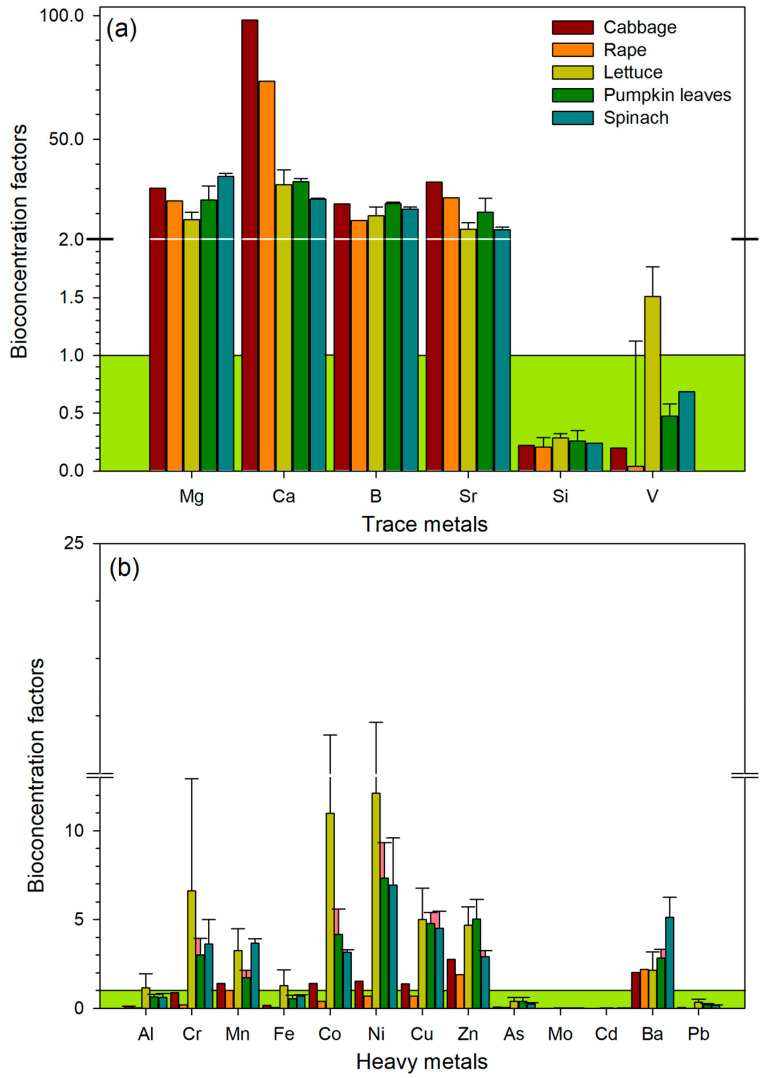
The bio-concentration factors (BCF) of trace metals (**a**) and heavy metals (**b**) in leafy edible vegetables from Tonga, South Africa. Red shade—BCF value greater than 1, suggesting a high metal translocation from soil to the vegetables; green shade—BCF values of less than 1, meaning that there is minimal translocation of metals from the soils to vegetables.

The BCF of trace metals exhibited the following trend: Ca > Sr > Mg > B > Si > V for trace metals (Figure 3a). Generally, the study indicated that all vegetables showed accumulation of trace metals, except Si and V, which showed low accumulation. However, the BCF values for V were accumulated in lettuce (Figure 3a). The BCF of heavy metals showed maximum translocations of Cr, Co, Mn, Ni, and Ba in lettuce, spinach, and pumpkin leaves. There was a maximum translocation of B in spinach and Cu in cabbage. Minimum translocations were observed in As, Mo, Cd, Pb, and Al from the soil to all leafy vegetables. (Figure 3b).

#### 3.3.2. Daily Intake of Metals (DIM)

**Figure 4:**
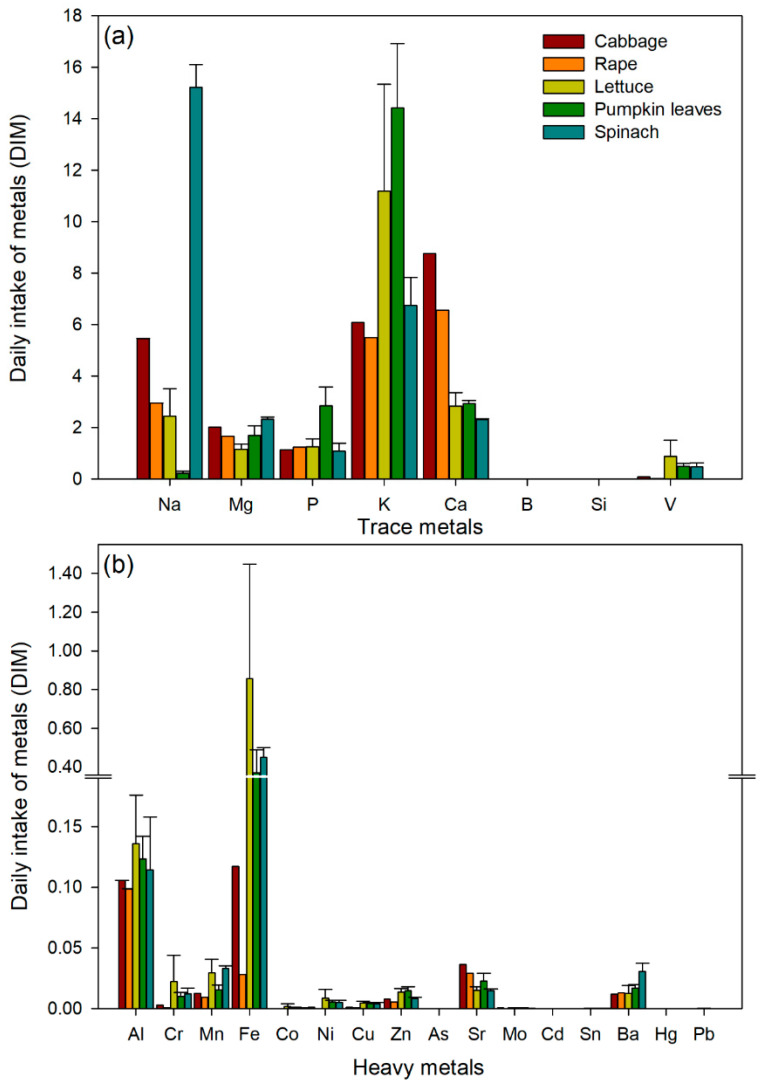
The daily intake of trace metals (**a**) and heavy metals (**b**) values from consuming leafy edible vegetables.

The daily intake of metals by humans when consuming leafy edible vegetables within the study area was compared to the RfD limits set by USEPA. A high intake of K was observed from the consumption of pumpkin leaves and lettuce and Na from the consumption of spinach. In the case of heavy metals, the highest intake of Fe was from lettuce, pumpkin leaves, and spinach (Figure 4). Our study showed that Fe exceeded the oral dose described by USEPA guidelines.

#### 3.3.3. Human Health Risk Index (HRI)

**Figure 5:**
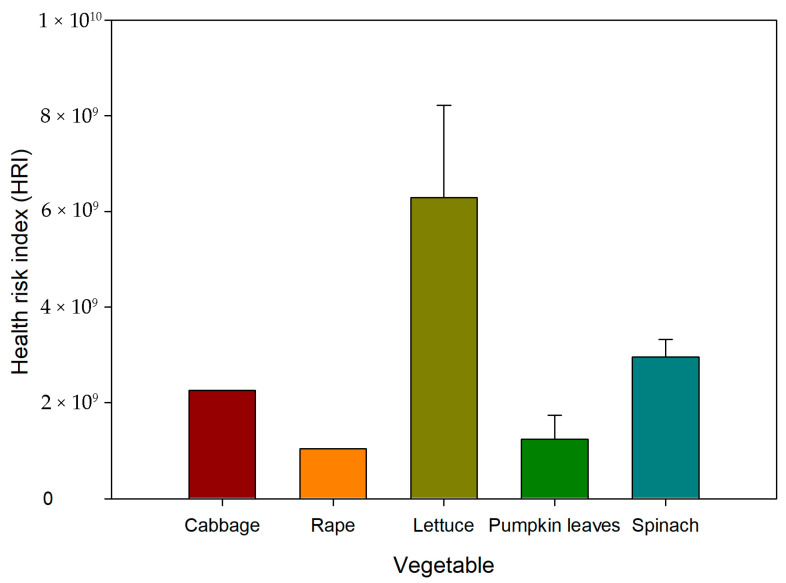
The risk to human health (HRI) by intake of metal through the consumption of vegetables. Red shade—the HRI value is equal to or higher than 1; it is therefore considered to be not safe for humans, as a potential health risk may occur. Green shade—the HRI ratio is lower than 1; the vegetables are therefore not likely to induce any health hazard to humans.

The HRI values for all vegetables were very high and in the following pattern: lettuce > spinach > cabbage > pumpkin leaves > rape (Figure 5). The study indicated all HRIs for all edible leafy vegetables were greater than 1, which indicated all vegetables accumulated metals that pose significant harm to humans.

#### 3.3.4. Target Hazard Quotient (THQ)

The THQ values for all trace metals (B, Si, and V) were greater than 1 for all vegetables. For heavy metals (Al, Cr, Mn, Fe, Ni, Zn, Cd, and Pb), THQ values were greater than 1 for all vegetables, whereas for Cu, As, Mo, Sn, and Ba in all edible leafy vegetables, the THQ values were less than 1; however, Ba was greater than 1 in spinach, with Co being less than 1 in rape and high in other vegetables (Table 4).

## 4. Discussion

The present study revealed varying concentrations of metals, with high concentrations of Na, Mg, K, and Ca recorded in soils. On the other hand, we found lower concentrations in P, V, and B. Variation in metal concentrations might be due to the natural processes and anthropogenic activities within the soils as [49], environmental changes affect the soil concentrations. These results align with a previous study that reported high concentrations of Na, K, Ca, and Mg in soils from fields where cabbage was cultivated [50]. 

Among the identified metals, Si had a high concentration in the soil, which might be a reason that Si has a limited uptake ability by plants and remains in the soil [51]. Silicon is found naturally in the soil [52] but is not beneficial for plant growth. However, it plays a major role in diseases and pest defense in the soil [53]. Potassium had a high mean concentration from the study area’s soils. Soils in the study area indicated that K uptake was more than other metals, such as Na, Ca and Mg. This has been confirmed by the findings from [54] that absorption of K can be 10 times greater than that of Na. Potassium availability in the soil is dependent on the pH of the soil and wastewaters [54]. 

For heavy metals, the high mean concentrations were found in Al and Fe only, and other metals such as Cr, Cu, Co, Ni, As, and Zn had low concentrations. Similar results were observed in agricultural fields where Fe [10] and Al had high concentrations [55]. On the contrary, high concentrations of Cr, Zn, Ni and Cu were observed in agricultural soils where different vegetables were grown [35]. These differences might be due to the activities, such as mining, fumes released from cars and even littering near the cultivation land. Our study reported higher concentrations of trace metals than the heavy metals in the soil, thus suggesting that the accumulation of these trace metals in the soil is an indication of the impact of fertilisers from agricultural activities, human activities and climate change.

Cabbage and rape leaves in the study area had high concentrations of Ca, K, Na, Mg, and P. These concentrations were consistent with the results reported by [56], where high concentrations of Ca, K, and P were observed and a high concentration of Na was also observed in cabbage [50]. Moreover, according to [56], cabbage had Si (2.1 mg kg^−1^), which was reported not to be a Si accumulator; however, our study reported that cabbage had accumulated about 377.2 mg kg^−1^ of Si. Potassium levels were higher in lettuce and pumpkin, as well as in all leafy vegetables, compared to Ca [57]. The current study found high Na accumulation in spinach, which was low in the leafy vegetables tested by Punchay et al. in Thailand. Overall, Mg was higher than B, Si, and Vi in all leafy vegetables, which is consistent with a study by [57].

Even if Mg is important for bone structure, metabolism, and as a powerful antioxidant, a high level of Mg can be harmful and affect the nervous system, which, in turn, may cause insomnia, depression, or illusions and, eventually, progressive alterations in gait and balance and tremors and Parkinson-like symptoms [58]. 

This study indicates that mean concentrations of Al and Fe were high in all heavy metals in all the vegetables. Similar results were reported by Gebeyehu and Bayissa [59] and Guadie et al. [60], where they observed leafy vegetables having a higher concentration of Fe than Cr, Cu, and Pb. Furthermore, lettuce was the highest accumulator of Fe compared to other vegetables. Contrarily, it was observed that cabbage was the highest accumulator of Fe [60]. Aluminium had higher mean concentrations in lettuce and spinach, Ghasemidehkordi et al. observed where spinach accumulated high concentrations of Al as compared to other investigated leafy vegetables [61].

The PCA axes 1 and 2 contributed 42.5% and 18.1% of the total variance, respectively, and showed high and moderate positive relations for Sr, Ca, Mg, Sn, and Zn. Zn had positive loadings, and most of the trace metals showed positive loadings. The current study reported that Cu, Cd, Pb, and Zn have a strong relationship with lettuce. This trend was also reported by Manzoor et al., who found that Cu, Cd, Pb, and Zn are strongly associated with lettuce [62].

The current study indicated that trace metals (Mg, Ca, B, Sr, and V in lettuce) had maximum translocation from the soil into all leafy vegetables, which indicated that their BCF values were greater than 1. Si and V had a minimal translocation from the soil to the leafy vegetables. Contrary to the study of Polechońska et al. [63], trace metals had a lower BCF, meaning that these metals had a passive translocation from the soil into the leafy vegetables. The maximum translocation of metals suggests that the tested vegetables may be useful in the phytoextraction of Mg, Ca, B, Sr, and V. The results of this study show that BCF values vary, and they have been shown to be species–dependent. The BCF values indicate whether the plant is an accumulator or an excluder [64]. Regarding heavy metals, the BCF showed maximum translocations of Cr, Co, Mn, Ni, and Ba in lettuce, spinach, and pumpkin leaves. There was a maximum translocation of B in spinach and Cu in cabbage. Minimum translocations were observed in As, Mo, Cd, Pb, and Al from the soil to all leafy vegetables. Similarly, Latif et al. [65] reported that Cd was passively translocated from the soil into pumpkin leaves, and contrarily, a high BCF for Cr was observed in spinach, however, Fontes et al. [66] observed Cd was highly translocated from the soil into lettuce. The translocation of metals from soils into the plant tissues depends on the soil properties, the vegetable’s ability to absorb metals and nutrient management [67]. Therefore, it is important to consider these factors when choosing vegetables for consumption, as the risk of human exposure to metal contamination can be considerably decreased. This study indicates that the tested leafy vegetables are accumulators of metals.

Daily intake of metals (DIM) is used to determine the exposure of metals being consumed by human beings [35]. There are possible human exposure routes, such as the food chain, skin contact, and inhalation, but oral intake is considered to be the main route for exposure in the food chain [68]. Our study indicated that through consuming pumpkin leaves or spinach around the study area, there is a chance of consuming high amounts of K and Na, respectively.

The study indicated that HRI for all edible leafy vegetables was greater than 1, which indicated all vegetables accumulated metals which pose significant harm to humans [69]. The high HRI values show the possibility of high BCF values of heavy metals being transferred from the soil to the vegetables [70]. The results suggest that the population is highly exposed to high health risks from the metals. 

The target hazard quotient method was used to measure the potential human health risk posed by metals through the consumption of vegetables [71]. The THQ values for all trace metals (B, Si and V) were greater than 1 for all vegetables. For heavy metals (Al, Cr, Mn, Fe, Ni, Zn, Cd, and Pb), THQ values were greater than 1 for all vegetables, whereas for Cu, As, Mo, Sn, and Ba in all edible leafy vegetables, the THQ values were less than 1; however, Ba was greater than 1 in spinach, with Co being less than 1 in rape and high in other vegetables. The current study is in agreement with results reported by Sharma et al. [72], where spinach was found to be more hazardous due to high THQ values greater than 1 of Cd, Co, Fe and Pb. Irrespective of the hazardous effects of these heavy metals, they have some important roles in human bodies; Co is found in vitamin B12 with various physiological functions [73]. Sharma et al. [72] further reported that these heavy metals have health hazards. They target some specific organs: Cd targets kidneys, Co targets the endocrine gland, and Cu and Fe target the gastrointestinal tract due to their high THQ values. If THQ values of less than 1 mean no non-carcinogenic risks and those greater than 1 indicate there is a considered potential of substantial health risk hazard [36], then, in the current study, we observed that these leafy vegetables pose a significant hazard to human beings due to high THQ values.

## 5. Conclusions

The study concludes that different vegetables accumulated different metals at different concentration levels. Furthermore, the study indicates potential health risks associated with consuming vegetables from the current study area due to the high accumulation of metals. There is a high translocation of metals from the soil into the vegetables. In addition, the results of the health risk assessment revealed that the HRI and THQ values through consumption of vegetables were >1, revealing that the people consuming these vegetables might suffer a higher non-carcinogenic risk. Thus, an extensive investigation on heavy metals in this area should be a priority since metal concentrations exceeded the limits guided by the WHO/FAO.

## Figures and Tables

**Table 1:** Concentration of metals (mean ± standard deviation) measured in soils (shaded in grey) and edible leafy vegetables from Tonga, South Africa.

Metal	Unit	Soil (*n* = 4)	Cabbage (*n* = 1)	Rape (*n* = 1)	Lettuce (*n* = 4)		Pumpkin (*n* = 4)		Spinach (*n* = 2)	
					Range	Mean ± SD	Range	Mean ± SD	Range	Mean ± SD
Na	mg/kg	344.6 ± 29.6	19,505.2	10,514.3	4350.9–13,537.1	8698.9 ± 3863	486.5–1106.1	799 ± 282.1	52,152–56,542.9	54,347.5± 3104.8
Mg	mg/kg	678.4 ± 237.3	7186.8	5945.6	3279–4936.1	4146.8 ± 714	4066.9–6952.7	6041.9 ± 1349.1	8122.4–8503.2	8312.8 ± 269.2
P	mg/kg	57.4 ± 11.6	4031.3	4409.5	2922–5616	447 ± 31,126	7413–13,689	10,182 ± 2598	3124–4672	3898 ± 1095
K	mg/kg	641.5 ± 67.8	21,746	19,603	25,270–60,077	39,940 ± 14,811	39,447–60,354	51,499 ± 8879	21,325–26,835	24,080 ± 3896
Ca	mg/kg	318.7 ± 29.2	31,268	23,448	8253–11,906	10,097 ± 1878	10,001–10,994	10,476 ± 409.2	8130–8323	8227 ± 136.5
B	mg/kg	1.4 ± 1.2	32.6	23.5	20.6–31.3	26.1 ± 4.7	32–33.7	32.8 ± 0.7	28.9–30.5	29.7 ± 1.1
Al	mg/kg	2753 ± 1301	328.6	93.3	675.1–6092	3165 ± 2233	1187–2075	1774 ± 410.3	1263–2108.3	1685 ± 598
Si	mg/kg	1706.5 ± 813.5	377.2	351.7	279.5–602.2	485.5 ± 142.4	386.9–538.6	439.3 ± 67.9	298.3–518.4	408.4 ± 155.6
V	mg/kg	6.7 ± 2.9	1.3	0.3	1.8–19.3	10.1 ± 7.3	1.8–5.7	3.1 ± 1.7	4.1–5	4.6 ± 0.7
Cr	mg/kg	12.1 ± 7.7	10.6	2.4	8.4–186.6	80.1 ± 76.1	21.1–47.9	36.3 ± 11.3	32–55.6	43.8 ± 16.6
Mn	mg/kg	32.3 ± 29.5	45.3	32.9	47.3–139.4	104.9 ± 39.8	46.9–75.9	55.8 ± 13.4	113.4–124.3	118.8 ± 7.6
Fe	mg/kg	2379 ± 1292	417.8	100.1	682–5764	3063 ± 2107	930.4–1928	1321 ± 427.4	1491–1737	1614 ± 174.3
Co	mg/kg	0.7 ± 0.4	0.9	0.3	0.7–17.2	7.2 ± 7	2–4.1	2.7 ± 0.9	2–2.1	2 ± 0.1
Ni	mg/kg	2.6 ± 2.2	3.9	1.8	4.4–63.5	31.3 ± 25.2	13.4–25.8	18.9 ± 5.1	13–22.8	17.9 ± 6.9
Cu	mg/kg	3.3 ± 1.4	4.5	2.3	7.9–20.4	16.3 ± 5.7	13.1–17.9	15.6 ± 1.9	12.4–16.9	14.7 ± 3.1
Zn	mg/kg	10.4 ± 7.4	28.8	19.9	34.6–58.9	48.7 ± 10.8	42.8–69.2	52.3 ± 11.6	27.9–32.7	30.3 ± 3.3
As	mg/kg	0.4 ± 0.14	0.03	0.02	0.08–0.31	0.17 ± 0.10	0.09–0.32	0.18 ± 0.10	0.09–0.13	0.11 ± 0.03
Sr	mg/kg	4.0 ± 2.8	130.3	104.8	45.5–67.9	54 ± 10.5	54–109.4	81.6 ± 22.8	50–56.6	53.3 ± 4.6
Mo	mg/kg	118.2 ± 61.4	3.1	1.6	0.2–2.4	1.1 ± 0.9	1.3–3	1.9 ± 0.7	1–1.1	1 ± 0
Cd	mg/kg	0.00 ± 0.00	0.04	0.01	0.09–0.17	0.11 ± 0.04	0.00–0.00	0.02 ± 0.01	0.05–0.06	0.05 ± 0.01
Sn	mg/kg	0.3 ± 0.1	1.5	1.6	0.8–1.5	1.2 ± 0.2	1.1–1.3	1.2 ± 0	1.4–1.5	1.4 ± 0.1
Ba	mg/kg	21.3 ± 12.6	42.9	46.7	15.9–66.7	45.8 ± 22.1	46–72.4	60.2 ± 11	91.9–126.2	109.1 ± 24.2
Hg	mg/kg	<0.01	0.00	0.01	0.00–0.00	0.01 ± 0.01	0.00–0.00	0.01 ± 0.00	0.01–0.00	0.01 ± 0.00
Pb	mg/kg	2.2 ± 0.9	0.1	0.1	0.3–1.3	0.7 ± 0.4	0.3–0.6	0.4 ± 0.1	0.3–0.4	0.3 ± 0

**Table 2:** Analysis of variance results for metal concentrations in leafy edible vegetables. Values in bold indicate significant differences at *p* < 0.05. Abbreviations: df—degrees of freedom.

Variable	Df	F	*p*
Trace metals			
Na	**4**	**45.548**	**<0.001**
Mg	**4**	**4.745**	**0.036**
P	**4**	**7.045**	**0.013**
K	**4**	**4.840**	**0.034**
Ca	**4**	**29.207**	**<0.001**
B	4	2.910	0.103
Si	4	0.299	0.870
V	4	2.866	0.106
Heavy metals			
Al	**4**	**6.515**	**0.016**
Cr	4	2.395	0.148
Mn	4	4.018	0.053
Fe	**4**	**5.954**	**0.021**
Co	4	1.594	0.277
Ni	4	2.406	0.147
Cu	**4**	**9.410**	**0.006**
Zn	**4**	**6.273**	**0.018**
As	**4**	**4.433**	**0.042**
Sr	**4**	**4.983**	**0.032**
Mo	4	1.566	0.283
Cd	**4**	**20.965**	**0.001**
Sn	4	1.250	0.373
Ba	4	1.786	0.236
Hg	4	0.304	0.867
Pb	4	2.962	0.100

**Table 3:** Principal component analysis (PCA) of trace and heavy metals for various vegetables.

Variance Extracted				
Axis	Eigen Value	% of Variance	Cumulative % of Variance	Broken-Stick Eigen Value
1	10.62	42.46	42.46	3.82
2	4.51	18.05	60.51	2.82
3	3.02	12.08	72.59	2.32
4	2.67	10.68	83.28	1.98
Eigen Vectors				
Metals	Axis 1	Axis 2	Axis 3	Axis 4
Trace metals				
Na	0.14	−0.70	0.56	−0.25
Mg	0.54	−0.14	0.31	0.11
P	0.14	0.86	0.18	0.09
K	−0.20	0.90	0.06	−0.18
Ca	0.59	−0.19	−0.26	0.59
Si	−0.40	0.14	−0.49	−0.11
V	−0.95	−0.19	−0.04	0.1
B	0.38	0.57	0.55	0
Heavy metals				
Al	−0.98	−0.01	0.08	0.13
Cr	−0.94	−0.15	0.02	0.28
Mn	−0.73	−0.43	0.4	−0.21
Fe	−0.97	−0.13	0.02	0.1
Co	−0.94	−0.12	−0.08	0.3
Ni	−0.95	0.00	0.06	0.23
Cu	−0.78	0.34	0.41	−0.22
Zn	−0.27	0.83	−0.13	−0.37
As	−0.76	0.40	0.15	0.2
Sr	0.60	0.22	−0.01	0.65
Mo	0.04	0.15	0.08	0.88
Cd	−0.41	−0.26	−0.49	−0.55
Sn	0.47	−0.48	−0.2	0.11
Sb	0.07	0.61	0.13	0.04
Ba	0.07	−0.26	0.93	−0.08
Hg	−0.78	−0.12	0.05	0.36
Pb	−0.99	0.06	−0.01	0.09

**Table 4:** The target hazard quotient (THQ) for the leafy edible vegetables. The bold and not bold numbers indicate the potential of substantial non-carcinogenic risks and no non-carcinogenic risks, respectively.

Metals	Cabbage	Rape	Lettuce	Pumpkin Leaves	Spinach
Trace metals					
B	**975.1**	**702.7**	**782.3 ± 143**	**984.1 ± 21.6**	**890.6 ± c3.3**
Si	**4476.3**	**4173.5**	**5762.2 ± 1690.6**	**5214.4 ± 806.8**	**4846.7 ± 1847.3**
V	**340.7**	**64.8**	**2576 ± 1853.2**	**809 ± 439**	**1167.7 ± 179.1**
Heavy metals					
Al	**670.2**	**190.2**	**6454.4 ± 4554.5**	**3618.4 ± 836.9**	**3437.1 ± 1219.6**
Cr	**118.1**	**27.2**	**891.2 ± 846.9**	**403.7 ± 126.1**	**487 ± 185.4**
Mn	**308.2**	**224.2**	**713.9 ± 271.4**	**380.3 ± 91.8**	**809.1 ± 52.2**
Fe	**2334.2**	**559.3**	**17,110.3 ± 11,768.7**	**7379.4 ± 2388**	**9015.7 ± 974**
Co	**1.1**	0.3	**8.8 ± 8.5**	**3.3 ± 1.1**	**2.5 ± 0.1**
Ni	**>10,000**	**>10,000**	**>10,000**	**>10,000**	**>10,000**
Cu	0.1	0.03	0.2 ± 0.2	0.2 ± 0.01	0.2 ± 0.01
Zn	**>10,000**	**>10,000**	**>10,000**	**>10,000**	**>10,000**
As	0.01	0.01	0.05 ± 0.03	0.05 ± 0.03	0.03 ± 0.01
Sr	**>10,000**	**>10,000**	**>10,000**	**>10,000**	**>10,000**
Mo	0.0001	0.0001	<0.0001	<0.0001	<0.0001
Cd	**>10,000**	**>10,000**	**>10,000**	**>10,000**	**>10,000**
Sn	0.0001	0.0001	<0.0001	<0.0001	<0.0001
Ba	0.5	0.5	0.5 ± 0.2	0.6 ± 0.1	**1.2 ± 0.2**
Pb	**659.6**	**338.4**	**4392.4 ± 2393.2**	**2660.4 ± 710.2**	**2300.8 ± 228.2**

## Data Availability

All data has been presented in the manuscript.
